# The Incidence and Severity of COVID-19 Infection Post Vaccination in Saudi Arabia

**DOI:** 10.7759/cureus.39766

**Published:** 2023-05-31

**Authors:** Yahya F Jamous, Mohamed Saleem T Sheik Uduman, Mohammed Alnakhli, Ahmed Alshaibi, Mohammad Alhawsawi, Amal Binsalman

**Affiliations:** 1 Vaccines and Bioprocessing, King Abdulaziz City for Science and Technology, Riyadh, SAU; 2 Pharmacy, Riyadh Elm University, Riyadh, SAU; 3 Medicine and Surgery, King Fahad Hospital, Madinah, SAU; 4 Medicine, Al-Rayan College, Madinah, SAU

**Keywords:** pfizer-biontech vaccine, pain, sars-cov-2, coronavirus, covid-19

## Abstract

Background

Severe acute respiratory syndrome coronavirus 2 (SARS-CoV-2) is the causative agent of coronavirus disease 2019 (COVID-19). Presently, there is ongoing continuous research for more therapeutic options with a wide variety of vaccine availability. However, many people have worried about the vaccine's side effects. Hence, the current study was conducted to determine the prevalence of vaccinated individuals, side effects, and the rate of infectivity post vaccination including the three doses of vaccinations.

Methods

A cross-sectional questionnaire-based survey was conducted using Google Forms (Google, Inc., Mountain View, CA). Five hundred forty-three individuals participated and reported their status of COVID-19 infection, vaccination, and side effects. All the participants from Saudi Arabia received all the vaccine shots including the booster dose.

Results

Most of the Saudi nationals were fully vaccinated, and most received Pfizer vaccines for their first and second shots. Pain at the injection site was reported as the most common adverse effect followed by fever, headache, fatigue, and joint pain.

Conclusion

From the findings, it is concluded that most of the population of Saudi Arabia was vaccinated effectively. Pain at the injection site is identified as the primary adverse effect of vaccination. Most of the population is vaccinated with the Pfizer vaccine. Long-term side effect monitoring is recommended with large population studies to confirm the status of vaccines and adverse effects.

## Introduction

Coronavirus disease 2019 (COVID-19) is a severe acute respiratory syndrome (SARS) caused by severe acute respiratory syndrome coronavirus 2 (SARS-CoV-2), a novel virus belonging to the Coronaviridae family [1,2]. This devastating disease has led to public health crises worldwide [2]. COVID-19 first emerged in Wuhan, China, in December 2019 before spreading to other countries [1]. On January 30, 2020, the World Health Organization (WHO) declared the virus outbreak a public health emergency of international concern and on March 11, 2020, classified it as a pandemic [3]. The COVID-19 pandemic has affected healthcare systems, national economies, and societal conditions, including individual lifestyles [4]. According to statistics and reports from reputable organizations, no fewer than 611 million cases were reported by September 23, 2022, with more than 6.512 million officially recorded deaths [5].

Non-pharmaceutical interventions can bolster control measures for COVID-19, and vaccine development is crucial for combating the virus and disease [6]. Before the COVID-19 vaccine was finalized and implemented, laboratories and pharmaceutical companies tested their vaccine samples on humans affected by COVID-19, deeming them safe and effective. Historical data reveals that vaccination programs have been widely adopted in many countries since December 2020 to protect their populations from the severe impacts of the virus. This effort approved four inactivated vaccines, two messenger RNA (mRNA) vaccines, two protein subunit vaccines, and three adenovirus vector vaccines [7]. By the end of 2020, several international regulators, including the Saudi FDA (SFDA), authorized three of these vaccines for emergency use [8].

Recent statistics indicate that over 818,000 confirmed COVID-19 cases were documented in Saudi Arabia, resulting in more than 9,000 deaths. As of September 26, 2022, vaccine doses were administered 67,839 times [9]. In December 2020, following the rapid approval of Pfizer-BioNTech's first mRNA vaccine (BNT162b2) by the FDA for emergency use, an immunization campaign was launched in Saudi Arabia. The analysis showed no major side effects after administering the first COVID-19 vaccine dose during the initial phase of the immunization program. However, the SFDA noted a correlation between the incidence of thrombosis with a low platelet count and the use of the Oxford/AstraZeneca vaccine [10]. Another study from Saudi Arabia evaluated the infection rate of SARS-CoV-2 among individuals who received a single dose of different vaccines, including Oxford/AstraZeneca (AZD1222) and Pfizer-BioNTech (BNT162b2) vaccines. Statistics indicated that 92% of vaccinated individuals remained uninfected within 3-8 months following vaccination [8]. However, a more comprehensive understanding of vaccination performance requires analyzing various populations. Consequently, collecting data from diverse populations before and after vaccination is essential. Real-world data and analysis offer insights into the effects of vaccination on different ethnic populations and subpopulations, which can be further categorized based on age, gender, occupation, comorbidities, and nationality [8]. This study aims to determine the prevalence of vaccinated individuals, side effects, and infection rates post vaccination, including the three doses of vaccinations.

## Materials and methods

Study setting and design

A cross-sectional internet questionnaire-based survey was conducted in various regions of Saudi Arabia during October and December 2022. Initially, the questionnaire was designed in English and translated into Arabic to make it easy for the public. All the questions in the questionnaire were divided into various sections such as demographic characteristics of the study participants, distribution of lifestyle risk factors, distribution of parameters on the vaccine, association of the vaccine and severity of the disease, and association of clinical parameters and disease infectivity. The King Abdulaziz City for Science and Technology Institutional Review Board issued approval IRB#22007. The questionnaire is valid until September 19, 2023.

Study sampling

All the participants were recruited by convenience sampling method through Google Forms (Google, Inc., Mountain View, CA) email and social media invitations. Instructions were made through invitation, the study is only for research purposes, and study participation was voluntary. To ensure the privacy of the participants, data-related personal information was not collected.

Inclusion/exclusion criteria

Males and females with a minimum age of 18 years or older, irrespective of their COVID-19 infection status and vaccination status, were included in the study. The participants with ages of less than 18 years and those that are not interested to participate in the study were excluded from the research.

Statistical analysis

Descriptive statistics were used to analyze the parameters by using Statistical Package for Social Sciences (SPSS) version 26 (IBM SPSS Statistics, Armonk, NY). All the categorical variables were presented as frequencies and percentages. The significance level of dependent variables has been assessed by paired t-test. The significance level for p-value will be considered as 0.05.

## Results

Table 1 presents the demographic distribution of the study participants. A majority of the participants (56.7%) were in the age group of 18-28 years. A notable proportion of the participants (288) were female. The majority of the participants (75.9%) were Saudi nationals. Among the participants, 48.3% had completed a bachelor's degree. The study population was almost equally divided between employed individuals (45.5%) and students (43.6%). Geographically, 42.2% of the participants resided in the western region of Saudi Arabia.

**Table 1:** Distribution of the participants as per demographic status (n=543)

Variables		n=543	
		Numbers	Percentage
Gender	Male	255	47
	Female	288	53
Age	18-28	308	56.7
	29-38	143	26.3
	39-48	61	11.2
	49-58	28	5.2
	>60	3	0.6
Nationality	Saudi	412	75.9
	Non-Saudi	131	24.1
Education	Less than high school	21	3.9
	High school	146	26.9
	Bachelor's	262	48.3
	Master's	93	17.1
	Doctorate	21	3.9
Employment	Employed	247	45.5
	Retired	9	1.7
	Self-employed	13	2.4
	Student	237	43.6
	Unemployed	37	6.8
Location	Eastern	127	23.4
	Middle	120	22.1
	Northern border	9	1.7
	Northern	41	7.6
	Southern	17	3.1
	Western	229	42.2

A total of 44.8% of the participants indicated no history of smoking, while 54.7% reported engaging in regular exercise. Among the respondents, 61.9% stated they had no comorbidities. Asthma emerged as the most prevalent comorbidity, affecting 22.7% of the participants. These results are presented in Table 2.

**Table 2:** Distribution of lifestyle risk factors among the study participants

Variables		n=543	
		Numbers	Percentage
History of smoking	Yes	184	33.9
	Occasionally	116	21.4
	No	243	44.8
Exercise	Yes	297	54.7
	No	246	45.3
Comorbidity	Asthma	123	22.7
	Chronic obstructive pulmonary disease (COPD)	8	1.5
	Diabetes mellitus	42	7.7
	Hypertension	20	3.7
	Thyroid	14	2.6
	None	336	61.9
History of surgery	Yes	186	34.3

In the study, 73.7% of the participants reported being fully vaccinated with Pfizer, receiving the first (52.9%), second (56.5%), and third (53.4%) doses. Furthermore, 37.6% of the participants received their second dose 4-5 weeks after the first, and 20.6% received their third dose 6-7 weeks after the second. These findings are detailed in Table 3.

**Table 3:** Status of vaccines among the study participants

Variables		n=543	
		Numbers	Percentage
Status of vaccination	Fully vaccinated	400	73.7
	Completed two doses	110	20.3
	Completed one doses	2	0.4
	None	31	5.7
First vaccine: type	AstraZeneca	139	25.6
	Moderna	84	15.5
	Pfizer	287	52.9
	Others	33	6.1
Second vaccine: type	AstraZeneca	98	18
	Moderna	104	19.2
	Pfizer	307	56.5
	Others	34	6.3
Third vaccine: type	AstraZeneca	128	23.6
	Moderna	55	10.1
	Pfizer	290	53.4
	Others	70	12.9
Duration for the second dose	4-5 weeks	204	37.6
	5-6 weeks	82	15.1
	6-7 weeks	69	12.7
	7-8 weeks	79	14.5
	8-12 weeks	43	7.9
	>12 weeks	66	12.2
Duration for the third dose	4-5 weeks	72	13.3
	5-6 weeks	101	18.6
	6-7 weeks	112	20.6
	7-8 weeks	108	19.9
	8-12 weeks	54	9.9
	>12 weeks	96	17.7

Table 4 illustrates the reported side effects of the COVID-19 vaccine. A majority of the participants noted pain at the injection site as the primary side effect. Common flu-like symptoms, such as fever, body pain, headache, and joint pain, were also frequently reported among participants. Table 5 reveals no significant difference in COVID-19 infection rates before and after vaccination. Most participants indicated that they had not contracted the COVID-19 virus following vaccination, with any subsequent infections generally being mild to moderate in severity, as shown in Table 6.

**Table 4:** Status of side effects due to the vaccine

Side effects	First dose		Second dose		Third dose	
	Number	Percentage	Number	Percentage	Number	Percentage
Pain at the injection site	395	72.7	304	56	344	63.4
Whole-body pain	16	2.9	9	1.7	47	8.7
Blurred vision	1	0.2	-	-	-	-
Cough	2	0.4	1	0.2	-	-
Fatigue	17	3.2	34	6.3	2	0.4
Fever	66	12.2	100	18.4	28	5.2
Headache	7	1.3	21	3.9	5	0.9
Joint pain	3	0.6	5	0.9	5	0.9
Muscle pain	1	0.2	-	-	-	-
Shortness of breath	1	0.2	-	-	-	-
Sleepiness	3	0.6	25	4.6	1	0.2
Depression	1	0.2	-	-		
Dizziness	4	0.7	1	0.2	2	0.4
Backache	-	-	1	0.2		
Bone pain	-	-	2	0.4	2	0.4
Chest tightness	-	-	1	0.2	-	-
Palpitations	-	-	1	0.2	-	-
Sore throat	-	-	1	0.2	-	-
Chills	-	-	-	-	1	0.2
Hypertension	-	-	-	-	1	0.2
Leg swelling	-	-	-	-	1	0.2
Stress and anxiety	-	-	-	-	10	1.8
Others	-	-	2	0.4	4	0.8
None	26	4.8	35	6.4	90	16.5

**Table 5:** Association of the vaccine and COVID-19 infection COVID-19: coronavirus disease 2019

Variables		Number	Percentage	P-value
COVID-19 infection before the vaccine	Yes	212	39	0.166
	No	331	61	
COVID-19 infection after the vaccine	Yes	236	43.5	
	No	307	56.5	

**Table 6:** Association of the vaccine and severity of the disease

Severity of infection after the vaccine		Number	Percentage
First dose	Mild	117	21.5
	Moderate	63	11.6
	Severe	55	10.1
	None	308	56.7
Second dose	Mild	59	10.9
	Moderate	99	18.2

## Discussion

A cross-sectional study was conducted to investigate the willingness and acceptance levels of vaccines and the occurrence of adverse effects among the Saudi population. Factors such as awareness, side effects, and religious and economic aspects [11] consistently influence the acceptance of the COVID-19 vaccine. As a result, a post-marketing assessment of vaccine side effects is crucial [12].

In the present study, 543 individuals participated, with 255 males (47%) and 288 females (53%). These findings align with other reports in which females represented a larger proportion of the participants [13,14]. Most of the participants (308, 56.7%) belonged to the age group of 18-28. Another study reported that 65.7% of the participants were below 60 years of age [14]. Saudi nationals comprised a significant portion of the participants (412, 75.9%), which parallels another study in which 93.4% of the participants were Saudi citizens [14]. A high percentage of the population (262, 48.3%) had completed a bachelor's degree compared to other education levels, a finding consistent with other reported data [13,14]. A total of 247 (45.5%) participants indicated employment, while 237 (43.6%) reported being students. In contrast, another study reported a higher percentage of employed individuals with a lower percentage of student participation [13]. In the present study, 229 (42.2%) participants were from the western region of Saudi Arabia, whereas a smaller percentage (1.7%) represented the northern region.

Most participants identified as nonsmokers (243, 44.8%) and reported exercising regularly (297, 54.7%). A total of 336 participants (61.9%) stated that they had no comorbidities, which is comparable to another study reporting that 73.2% of the participants were healthy [13]. Asthma was noted as a comorbidity among 22.7% of the participants. Furthermore, 357 (65.7%) participants indicated no surgical history.

Among the 543 participants, 400 reported being fully vaccinated, while 31 indicated that they were unvaccinated for COVID-19. A substantial proportion of the participants received the Pfizer vaccine for their first (52.9%), second (56.5%), and third (53.4%) doses. Another study reported that 100% of the population received the Pfizer vaccine [14]. Following Pfizer, the majority of the participants received their first (25.6%, AstraZeneca), second (19.2%, Moderna), and third (23.6%, AstraZeneca) vaccines. Most participants (37.6%) received their second vaccine dose 4-5 weeks after the first dose and their third (20.6%) 6-7 weeks after the second dose.

Pain at the injection site was the most commonly reported side effect for the first (72.7%), second (56%), and third (63.4%) doses. In a related study, 42.2% of the participants who received the Sinopharm vaccine reported similar side effects [13,14]. According to the data, side effects, such as pain at the injection site, chills, headache, fatigue, and fever, were more prevalent across all three vaccine doses [15].

Of the 543 participants, 212 (39%) reported contracting the COVID-19 virus before vaccination, which is higher than other reports showing that only 4.2% of the population was infected before vaccination [14]. Post vaccination, 236 (43.5%) participants reported contracting the virus. A considerable number of the participants reported contracting COVID-19 after the first (46%), second (41.2%), and third (12.8%) vaccine doses. The infection severity was mild (21.5%), moderate (18.2%), and mild (14%) after the first, second, and third doses, respectively. Among the vaccinated and unvaccinated populations, the majority (45.3% and 19.7%) of infected individuals underwent home isolation treatments. COVID-19-infected individuals, both vaccinated and unvaccinated, were admitted to the hospital for 1-2 weeks.

This study is one of the critical investigations illuminating the side effects of the COVID-19 vaccine. Only a few studies have documented the post-marketing assessment of vaccine side effects, most of which primarily focus on the first two Pfizer vaccine doses. Our study emphasizes complete vaccination with various vaccine types administered to the participants. The current study's limitations include focusing on the short-term side effects of vaccines taken by the Saudi Arabian population and a relatively small number of participants.

## Conclusions

In conclusion, this study's results demonstrate that most of Saudi Arabia's population has been effectively vaccinated against COVID-19. The primary adverse effect reported was pain at the injection site. The Pfizer vaccine was administered to most individuals in the study population. It is recommended that long-term side effect monitoring be conducted through extensive population studies to ascertain the vaccine's safety and potential adverse effects.

## Appendices

The questionnaire is shown in https://emea01.safelinks.protection.outlook.com/?url=https%3A%2F%2Fdocs.google.com%2Fforms%2Fd%2Fe%2F1FAIpQLSdz4JWCMKxuMdBsK9IwnTduFoLkjbPNiGxd_q0NimTuERlS5A%2Fviewform&data=05%7C01%7C%7C435e01d99cc2423ee7be08db5f5ae497%7C84df9e7fe9f640afb435aaaaaaaaaaaa%7C1%7C0%7C638208616200025600%7CUnknown%7CTWFpbGZsb3d8eyJWIjoiMC4wLjAwMDAiLCJQIjoiV2luMzIiLCJBTiI6Ik1haWwiLCJXVCI6Mn0%3D%7C3000%7C%7C%7C&sdata=SHAXJmGRVrHThPqIyOpCcoDkJVYd%2B8Fex2snZ05jFM0%3D&reserved=0.
